# Supplementary material for: An open-source framework for large-scale, flexible evaluation of biomedical text mining systems
Source: J Biomed Discov Collab. 2008 Jan 29;3:1. doi: 10.1186/1747-5333-3-1 (PMC2276192; doi:10.1186/1747-5333-3-1)
Supplement: Additional file 1 — Evaluation platform source code. The additional file contains the software evaluation framework discussed in this paper. The most current release can be downloaded from SourceForge.net [1]. See the accompanying README file for instructions on installation and use. Also included in the distribution is a collection of UIMA wrappers for some commonly used BioNLP tools and annotated corpora. [file 1747-5333-3-1-S1.zip › bionlp_uima_repository-1.0.1/docs/api/src-html/edu/uchsc/ccp/util/nlp/parser/corpus/IProLinkParser.html]

```
001    package edu.uchsc.ccp.util.nlp.parser.corpus;
002    
003    import java.io.BufferedReader;
004    import java.io.IOException;
005    import java.util.ArrayList;
006    import java.util.List;
007    import java.util.regex.Matcher;
008    import java.util.regex.Pattern;
009    
010    import edu.uchsc.ccp.util.nlp.GenericTextUtils;
011    import edu.uchsc.ccp.util.nlp.annotation.AnnotationSet;
012    import edu.uchsc.ccp.util.nlp.annotation.Annotator;
013    import edu.uchsc.ccp.util.nlp.annotation.TextAnnotation;
014    import edu.uchsc.ccp.util.nlp.ei.xml.XMLEntityExtractor;
015    import edu.uchsc.ccp.util.nlp.mention.ClassMentionTypes;
016    
017    /**
018     * This class contains methods to parse the 2 IProLink XML corpora files. Specifically, this class is usefull if you want to process the corpus all at
019     * once, and then retrieve all of the annotations. Use the other classes in this package to process the documents one at a time.
020     * 
021     * @author williamb
022     * 
023     */
024    public class IProLinkParser {
025    
026        /* allBio1Documents holds [id, text] pairs for the documents in the collection */
027        private static List<String[]> allDocuments;
028    
029        private static List<TextAnnotation> allAnnotations;
030    
031        private static final String PROTEIN_TAG = "protein";
032    
033        private static final String ACRONYM_TAG = "acronym";
034    
035        private static final String COMPOUND_PROTEIN_TAG = "compound-protein";
036    
037        private static final String LONG_FORM_PROTEIN_TAG = "long_form";
038    
039        private static final int iProLinkCorpus_1_documentCollectionID = 9;
040    
041        private static final int iProLinkCorpus_2_documentCollectionID = 9;
042    
043        /**
044         * corpus1.xml has tags: protein, acronym, and compound-protein
045         * 
046         * @param iProLinkCorpus1File
047         * @param connectionPropertiesFile
048         * @throws IOException
049         */
050        public static void parseIProLinkCorpus1(BufferedReader br) throws IOException {
051            /* initialize document and annotation storage */
052            allAnnotations = new ArrayList<TextAnnotation>();
053            allDocuments = new ArrayList<String[]>();
054    
055            /* initialize annotator and annotationSet */
056            Annotator annotator = new Annotator(new Integer(11), "", "iProLink", "PIR");
057            AnnotationSet annotationSet = new AnnotationSet(new Integer(8), "iProLink prot/acr/cmpd Gold Annotations",
058                    "<protein>, <acronym>, and <compound-protein> annotations extracted from the iProLink corpus1.xml file.");
059    
060            // BufferedReader br = GenericTextUtils.openReader(iProLinkCorpus1File);
061            String line;
062    
063            Pattern idPattern = Pattern.compile("PMID:(\\d+)");
064            Pattern titlePattern = Pattern.compile("TI\\s+-\\s?(.*)");
065            Pattern abstractPattern = Pattern.compile("AB\\s+-\\s?(.*)");
066            Matcher m;
067    
068            String titleText = null;
069            String abstractText = null;
070            String id = null;
071            boolean inAbstract = false;
072            boolean inTitle = false;
073    
074            while ((line = br.readLine()) != null) {
075                // System.err.println("DEBUG -- IProLinkParser: LINE: " + line);
076                if (line.startsWith("PMID")) {
077                    // System.err.println("DEBUG -- IProLinkParser: FOUND ID LINE");
078                    m = idPattern.matcher(line);
079                    if (m.find()) {
080                        id = m.group(1);
081                        // System.err.println("DEBUG -- IProLinkParser: FOUND ID ***************************************");
082                    } else {
083                        System.err.println("WARNING -- Bio1Parser: ID expected but not found.");
084                    }
085                } else if (line.startsWith("TI  - ")) {
086                    // System.err.println("DEBUG -- IProLinkParser: FOUND TITLE LINE");
087                    m = titlePattern.matcher(line);
088                    if (m.find()) {
089                        titleText = m.group(1);
090                        // System.err.println("DEBUG -- IProLinkParser: FOUND TITLE ***************************************");
091                        inTitle = true;
092                        inAbstract = false;
093                    } else {
094                        System.err.println("WARNING -- Bio1Parser: Title expected but not found.");
095                    }
096                } else if (line.startsWith("AB  - ")) {
097                    // System.err.println("DEBUG -- IProLinkParser: FOUND ABSTRACT LINE");
098                    m = abstractPattern.matcher(line);
099                    if (m.find()) {
100                        abstractText = m.group(1);
101                        // System.err.println("DEBUG -- IProLinkParser: FOUND ABSTRACT ***************************************");
102                        inAbstract = true;
103                        inTitle = false;
104                    } else {
105                        System.err.println("WARNING -- Bio1Parser: Abstract expected but not found.");
106                    }
107                } else if ((line.startsWith(">") || line.trim().startsWith("</articleinfo>")) && (id != null)) {
108                    // System.err.println("DEBUG -- IProLinkParser: FOUND END OF ABSTRACT LINE");
109                    inAbstract = false;
110                    inTitle = false;
111    
112                    System.out.println("ID: " + id);
113                    System.out.println("TITLE: " + titleText);
114                    System.out.println("ABSTRACT: " + abstractText);
115    
116                    /* search the titlefor entities */
117                    String documentID = id + "-t";
118    
119                    String titleWithProteins = titleText.replaceAll("</?" + ACRONYM_TAG + ">", "").replaceAll(
120                            "</?" + COMPOUND_PROTEIN_TAG + ">", "");
121                    String titleWithAcronyms = titleText.replaceAll("</?" + PROTEIN_TAG + ">", "").replaceAll(
122                            "</?" + COMPOUND_PROTEIN_TAG + ">", "");
123                    String titleWithCompoundProteins = titleText.replaceAll("</?" + PROTEIN_TAG + ">", "").replaceAll(
124                            "</?" + ACRONYM_TAG + ">", "");
125    
126                    List<TextAnnotation> proteinAnnotations = XMLEntityExtractor.parseEntitiesFromSparseUntokenizedXML(titleWithProteins,
127                            PROTEIN_TAG, PROTEIN_TAG, documentID, iProLinkCorpus_1_documentCollectionID, ClassMentionTypes.PROTEIN, annotator,
128                            annotationSet);
129                    List<TextAnnotation> acronymAnnotations = XMLEntityExtractor.parseEntitiesFromSparseUntokenizedXML(titleWithAcronyms,
130                            ACRONYM_TAG, ACRONYM_TAG, documentID, iProLinkCorpus_1_documentCollectionID, ClassMentionTypes.PROTEIN_ACRONYM,
131                            annotator, annotationSet);
132                    List<TextAnnotation> compoundProteinAnnotations = XMLEntityExtractor.parseEntitiesFromSparseUntokenizedXML(
133                            titleWithCompoundProteins, COMPOUND_PROTEIN_TAG, COMPOUND_PROTEIN_TAG, documentID,
134                            iProLinkCorpus_1_documentCollectionID, ClassMentionTypes.PROTEIN_COMPOUND, annotator, annotationSet);
135    
136                    /* add all title annotations to the allBio1Annotations list */
137                    allAnnotations.addAll(proteinAnnotations);
138                    allAnnotations.addAll(acronymAnnotations);
139                    allAnnotations.addAll(compoundProteinAnnotations);
140    
141                    /* store the untagged title text */
142                    /* remove html tags from the title text */
143                    titleText = titleText.replaceAll(GenericTextUtils.escapeCharacterForRegEx("<" + PROTEIN_TAG + ">"), "").replaceAll(
144                            GenericTextUtils.escapeCharacterForRegEx("</" + PROTEIN_TAG + ">"), "");
145                    titleText = titleText.replaceAll(GenericTextUtils.escapeCharacterForRegEx("<" + ACRONYM_TAG + ">"), "").replaceAll(
146                            GenericTextUtils.escapeCharacterForRegEx("</" + ACRONYM_TAG + ">"), "");
147                    titleText = titleText.replaceAll(GenericTextUtils.escapeCharacterForRegEx("<" + COMPOUND_PROTEIN_TAG + ">"), "")
148                            .replaceAll(GenericTextUtils.escapeCharacterForRegEx("</" + COMPOUND_PROTEIN_TAG + ">"), "");
149    
150                    // System.err.println("DEBUG -- IProLinkParser: Found annotations in title: protein" + proteinAnnotations.size() + " acronym: " +
151                    // acronymAnnotations.size() + " compound: " + compoundProteinAnnotations.size());
152    
153                    String[] t = new String[2];
154                    t[0] = documentID;
155                    t[1] = titleText;
156                    allDocuments.add(t);
157    
158                    /* now search the abstract */
159                    documentID = id + "-a";
160    
161                    String abstractWithProteins = abstractText.replaceAll("</?" + ACRONYM_TAG + ">", "").replaceAll(
162                            "</?" + COMPOUND_PROTEIN_TAG + ">", "");
163                    String abstractWithAcronyms = abstractText.replaceAll("</?" + PROTEIN_TAG + ">", "").replaceAll(
164                            "</?" + COMPOUND_PROTEIN_TAG + ">", "");
165                    String abstractWithCompoundProteins = abstractText.replaceAll("</?" + PROTEIN_TAG + ">", "").replaceAll(
166                            "</?" + ACRONYM_TAG + ">", "");
167    
168                    proteinAnnotations = XMLEntityExtractor
169                            .parseEntitiesFromSparseUntokenizedXML(abstractWithProteins, PROTEIN_TAG, PROTEIN_TAG, documentID,
170                                    iProLinkCorpus_1_documentCollectionID, ClassMentionTypes.PROTEIN, annotator, annotationSet);
171                    acronymAnnotations = XMLEntityExtractor.parseEntitiesFromSparseUntokenizedXML(abstractWithAcronyms, ACRONYM_TAG,
172                            ACRONYM_TAG, documentID, iProLinkCorpus_1_documentCollectionID, ClassMentionTypes.PROTEIN_ACRONYM, annotator,
173                            annotationSet);
174                    compoundProteinAnnotations = XMLEntityExtractor.parseEntitiesFromSparseUntokenizedXML(abstractWithCompoundProteins,
175                            COMPOUND_PROTEIN_TAG, COMPOUND_PROTEIN_TAG, documentID, iProLinkCorpus_1_documentCollectionID,
176                            ClassMentionTypes.PROTEIN_COMPOUND, annotator, annotationSet);
177    
178                    // System.err.println("DEBUG -- IProLinkParser: Found annotations in abstract: protein" + proteinAnnotations.size() + " acronym: " +
179                    // acronymAnnotations.size() + " compound: " + compoundProteinAnnotations.size());
180    
181                    /* add all title annotations to the allBio1Annotations list */
182                    allAnnotations.addAll(proteinAnnotations);
183                    allAnnotations.addAll(acronymAnnotations);
184                    allAnnotations.addAll(compoundProteinAnnotations);
185    
186                    /* store the untagged abstract text */
187                    /* remove html tags from the abstract text */
188                    abstractText = abstractText.replaceAll(GenericTextUtils.escapeCharacterForRegEx("<" + PROTEIN_TAG + ">"), "").replaceAll(
189                            GenericTextUtils.escapeCharacterForRegEx("</" + PROTEIN_TAG + ">"), "");
190                    abstractText = abstractText.replaceAll(GenericTextUtils.escapeCharacterForRegEx("<" + ACRONYM_TAG + ">"), "").replaceAll(
191                            GenericTextUtils.escapeCharacterForRegEx("</" + ACRONYM_TAG + ">"), "");
192                    abstractText = abstractText.replaceAll(GenericTextUtils.escapeCharacterForRegEx("<" + COMPOUND_PROTEIN_TAG + ">"), "")
193                            .replaceAll(GenericTextUtils.escapeCharacterForRegEx("</" + COMPOUND_PROTEIN_TAG + ">"), "");
194    
195                    String[] a = new String[2];
196                    a[0] = documentID;
197                    a[1] = abstractText;
198                    allDocuments.add(a);
199    
200                } else {
201                    /* there are line breaks in the abstract, so if we are in the abstract, we add the line to the abstract text */
202                    if (inAbstract) {
203                        abstractText += (" " + line.trim());
204                    } else if (inTitle) {
205                        titleText += (" " + line.trim());
206                    } else {
207                        System.err.println("WARNING -- IProLinkParser: Unexpected tag at beginning of line: " + line);
208                    }
209                }
210            }
211    
212        }
213    
214        /**
215         * corpus2.xml has tags: protein and long-form
216         * 
217         * @param iProLinkCorpus2File
218         * @param connectionPropertiesFile
219         * @throws IOException
220         */
221        public static void parseIProLinkCorpus2(BufferedReader br) throws IOException {
222            /* initialize document and annotation storage */
223            allAnnotations = new ArrayList<TextAnnotation>();
224            allDocuments = new ArrayList<String[]>();
225    
226            /* initialize annotator and annotationSet */
227            Annotator annotator = new Annotator(new Integer(11), "", "iProLink", "PIR");
228            AnnotationSet annotationSet = new AnnotationSet(new Integer(9), "iProLink prot/long Gold Annotations",
229                    "<protein>, <long_form> annotations extracted from the iProLink corpus2.xml file.");
230    
231            // BufferedReader br = GenericTextUtils.openReader(iProLinkCorpus2File);
232            String line;
233    
234            Pattern idPattern = Pattern.compile("PMID:(\\d+)");
235            Pattern titlePattern = Pattern.compile("TI\\s+-\\s?(.*)");
236            Pattern abstractPattern = Pattern.compile("AB\\s+-\\s?(.*)");
237            Matcher m;
238    
239            String titleText = null;
240            String abstractText = null;
241            String id = null;
242            boolean inAbstract = false;
243            boolean inTitle = false;
244    
245            while ((line = br.readLine()) != null) {
246                if (line.startsWith("PMID")) {
247                    m = idPattern.matcher(line);
248                    if (m.find()) {
249                        id = m.group(1);
250                    } else {
251                        System.err.println("WARNING -- IProLink: ID expected but not found.");
252                    }
253                } else if (line.startsWith("TI  - ")) {
254                    m = titlePattern.matcher(line);
255                    if (m.find()) {
256                        titleText = m.group(1);
257                        inTitle = true;
258                        inAbstract = false;
259                    } else {
260                        System.err.println("WARNING -- IProLink: Title expected but not found.");
261                    }
262                } else if (line.startsWith("AB  - ")) {
263                    m = abstractPattern.matcher(line);
264                    if (m.find()) {
265                        abstractText = m.group(1);
266                        inAbstract = true;
267                        inTitle = false;
268                    } else {
269                        System.err.println("WARNING -- IProLink: Abstract expected but not found.");
270                    }
271                } else if (line.startsWith("</DOC>")) {
272                    inAbstract = false;
273                    inTitle = false;
274    
275                    System.out.println("ID: " + id);
276                    System.out.println("TITLE: " + titleText);
277                    System.out.println("ABSTRACT: " + abstractText);
278    
279                    /* search the titlefor entities */
280                    String documentID = id + "-t";
281    
282                    String titleWithProteins = titleText.replaceAll("</?" + LONG_FORM_PROTEIN_TAG + ">", "");
283                    String titleWithLongForms = titleText.replaceAll("</?" + PROTEIN_TAG + ">", "");
284    
285                    List<TextAnnotation> proteinAnnotations = XMLEntityExtractor.parseEntitiesFromSparseUntokenizedXML(titleWithProteins,
286                            PROTEIN_TAG, PROTEIN_TAG, documentID, iProLinkCorpus_2_documentCollectionID, ClassMentionTypes.PROTEIN, annotator,
287                            annotationSet);
288                    List<TextAnnotation> longFormAnnotations = XMLEntityExtractor.parseEntitiesFromSparseUntokenizedXML(titleWithLongForms,
289                            LONG_FORM_PROTEIN_TAG, LONG_FORM_PROTEIN_TAG, documentID, iProLinkCorpus_2_documentCollectionID,
290                            ClassMentionTypes.PROTEIN_LONG_FORM, annotator, annotationSet);
291    
292                    /* add all title annotations to the allBio1Annotations list */
293                    allAnnotations.addAll(proteinAnnotations);
294                    allAnnotations.addAll(longFormAnnotations);
295    
296                    /* store the untagged title text */
297                    /* remove html tags from the title text */
298                    titleText = titleText.replaceAll(GenericTextUtils.escapeCharacterForRegEx("<" + PROTEIN_TAG + ">"), "").replaceAll(
299                            GenericTextUtils.escapeCharacterForRegEx("</" + PROTEIN_TAG + ">"), "");
300                    titleText = titleText.replaceAll(GenericTextUtils.escapeCharacterForRegEx("<" + LONG_FORM_PROTEIN_TAG + ">"), "")
301                            .replaceAll(GenericTextUtils.escapeCharacterForRegEx("</" + LONG_FORM_PROTEIN_TAG + ">"), "");
302    
303                    String[] t = new String[2];
304                    t[0] = documentID;
305                    t[1] = titleText;
306                    allDocuments.add(t);
307    
308                    /* now search the abstract */
309                    documentID = id + "-a";
310    
311                    String abstractWithProteins = abstractText.replaceAll("</?" + LONG_FORM_PROTEIN_TAG + ">", "");
312                    String abstractWithLongForms = abstractText.replaceAll("</?" + PROTEIN_TAG + ">", "");
313    
314                    proteinAnnotations = XMLEntityExtractor
315                            .parseEntitiesFromSparseUntokenizedXML(abstractWithProteins, PROTEIN_TAG, PROTEIN_TAG, documentID,
316                                    iProLinkCorpus_2_documentCollectionID, ClassMentionTypes.PROTEIN, annotator, annotationSet);
317                    longFormAnnotations = XMLEntityExtractor.parseEntitiesFromSparseUntokenizedXML(abstractWithLongForms,
318                            LONG_FORM_PROTEIN_TAG, LONG_FORM_PROTEIN_TAG, documentID, iProLinkCorpus_2_documentCollectionID,
319                            ClassMentionTypes.PROTEIN_LONG_FORM, annotator, annotationSet);
320    
321                    /* add all title annotations to the allBio1Annotations list */
322                    allAnnotations.addAll(proteinAnnotations);
323                    allAnnotations.addAll(longFormAnnotations);
324    
325                    /* store the untagged abstract text */
326                    /* remove html tags from the abstract text */
327                    abstractText = abstractText.replaceAll(GenericTextUtils.escapeCharacterForRegEx("<" + PROTEIN_TAG + ">"), "").replaceAll(
328                            GenericTextUtils.escapeCharacterForRegEx("</" + PROTEIN_TAG + ">"), "");
329                    abstractText = abstractText.replaceAll(GenericTextUtils.escapeCharacterForRegEx("<" + LONG_FORM_PROTEIN_TAG + ">"), "")
330                            .replaceAll(GenericTextUtils.escapeCharacterForRegEx("</" + LONG_FORM_PROTEIN_TAG + ">"), "");
331    
332                    String[] a = new String[2];
333                    a[0] = documentID;
334                    a[1] = abstractText;
335                    allDocuments.add(a);
336    
337                } else {
338                    /* there are line breaks in the abstract, so if we are in the abstract, we add the line to the abstract text */
339                    if (inAbstract) {
340                        abstractText += (" " + line.trim());
341                    } else if (inTitle) {
342                        titleText += (" " + line.trim());
343                    } else {
344                        System.err.println("WARNING -- IProLink: Unexpected tag at beginning of line: " + line);
345                    }
346                }
347            }
348    
349        }
350    
351    //    public static void storeResults(int corpusIdentifier) throws IOException {
352    //        /* now store results in the database */
353    //        /* now add documents to the database */
354    //        for (String[] idTextPair : allDocuments) {
355    //            String documentID = idTextPair[0];
356    //            String documentText = idTextPair[1];
357    //
358    //            documentText = documentText.replaceAll("'", "''");
359    //
360    //            char TorA = documentID.charAt(documentID.length() - 1);
361    //
362    //            String sqlStr = "INSERT INTO IPROLINK_CORPUS VALUES ('" + documentID + "', '" + Character.toUpperCase(TorA) + "', "
363    //                    + corpusIdentifier + ", '" + documentText + "')";
364    //            System.out.println("SQLSTR: " + sqlStr);
365    //            // Database_Util.execute( AnnotationDBProperties.CONNECTION_PROPERTIES_FILE, sqlStr);
366    //        }
367    //
368    //        String sqlldrFileName = "iProLink" + corpusIdentifier + "_Annotations.sqlldr";
369    //        String runLoadFileName = "load_iProLink" + corpusIdentifier + "Annotations.sh";
370    //
371    //        /* add the annotations to the database in the form of an sqlldr file */
372    //        /* create the sqlldr input file */
373    //        PrintStream sqlldrFileStream = GenericTextUtils.openPrintStream(AnnotationDBProperties.EXTERNAL_BASE_ANNOTATION_DIR
374    //                + "/inputFiles/" + sqlldrFileName);
375    //        AnnotationDB_Util.printTextAnnotationListToSqlldrFile(sqlldrFileStream, allAnnotations, new ArrayList<String>(), true,
376    //                AnnotationDBProperties.CONNECTION_PROPERTIES_FILE, false);
377    //        sqlldrFileStream.close();
378    //
379    //        /* add the parsed gene annotations to the database */
380    //        AnnotationDB_Util.loadAnnotationsIntoDB(AnnotationDBProperties.EXTERNAL_BASE_ANNOTATION_DIR,
381    //                AnnotationDBProperties.LOCAL_BASE_ANNOTATION_DIR, sqlldrFileName, AnnotationDBProperties.SQLLDR_CONTROL_FILE_TEMPLATE,
382    //                runLoadFileName, AnnotationDBProperties.ANNOTATION_DB_STATUS_FLAG_FILE, true);
383    //    }
384    
385        public static List<TextAnnotation> getTextAnnotations() {
386            return allAnnotations;
387        }
388    
389        public static List<String[]> getDocuments() {
390            return allDocuments;
391        }
392    
393        /**
394         * 
395         * args[0] = iProLink corpus1 file<br>
396         * args[1] = iProLink corpus2 file<br>
397         * 
398         * @param args
399         */
400        public static final void main(String[] args) {
401            try {
402                // BufferedReader corpus1Reader = GenericTextUtils.openReader(args[0]);
403                // IProLinkParser.parseIProLinkCorpus1(corpus1Reader);
404                // corpus1Reader.close();
405                // IProLinkParser.storeResults(1);
406    
407                BufferedReader corpus2Reader = GenericTextUtils.openReader(args[1]);
408                IProLinkParser.parseIProLinkCorpus2(corpus2Reader);
409                corpus2Reader.close();
410    //            IProLinkParser.storeResults(2);
411            } catch (IOException e) {
412                e.printStackTrace();
413            }
414        }
415    
416    }
```
